# Supplementary material for: HIVprotI: an integrated web based platform for prediction and design of HIV proteins inhibitors
Source: J Cheminform. 2018 Mar 9;10:12. doi: 10.1186/s13321-018-0266-y (PMC5845081; doi:10.1186/s13321-018-0266-y)
Supplement: Supplementary file 2 — Additional file 2. Source code of HIVProtI web server. [file 13321_2018_266_MOESM2_ESM.zip › HIVprotI_Source-code/hivprotI/weka-3-6-6/doc/weka/filters/unsupervised/attribute/package-frame.html]

weka.filters.unsupervised.attribute


weka.filters.unsupervised.attribute

Classes 
  
AbstractTimeSeries
  
Add
  
AddCluster
  
AddExpression
  
AddID
  
AddNoise
  
AddValues
  
Center
  
ChangeDateFormat
  
ClassAssigner
  
ClusterMembership
  
Copy
  
Discretize
  
FirstOrder
  
InterquartileRange
  
KernelFilter
  
MakeIndicator
  
MathExpression
  
MergeTwoValues
  
MultiInstanceToPropositional
  
NominalToBinary
  
NominalToString
  
Normalize
  
NumericCleaner
  
NumericToBinary
  
NumericToNominal
  
NumericTransform
  
Obfuscate
  
PartitionedMultiFilter
  
PKIDiscretize
  
PotentialClassIgnorer
  
PrincipalComponents
  
PropositionalToMultiInstance
  
RandomProjection
  
RandomSubset
  
RELAGGS
  
Remove
  
RemoveType
  
RemoveUseless
  
Reorder
  
ReplaceMissingValues
  
Standardize
  
StringToNominal
  
StringToWordVector
  
SwapValues
  
TimeSeriesDelta
  
TimeSeriesTranslate
  
Wavelet
